# Ekbom Syndrome in the context of psychotic depression and cocaine use: A case report

**DOI:** 10.1192/j.eurpsy.2025.974

**Published:** 2025-08-26

**Authors:** C. P. Murta, F. Silva, L. Kobayashi

**Affiliations:** 1 Psychiatry, ULS Algarve, Faro, Portugal

## Abstract

**Introduction:**

Ekbom syndrome, also known as delusional infestation (DI), is a rare mono-symptomatic psychosis in which the patient has the firm and unshakable belief that one’s body, mainly their skin, is infested by parasites or bugs. DI primarily affects middle-aged women and it can be classified as primary or secondary, often associated with other psychiatric conditions like psychotic depression, or induced by substances such as cocaine or amphetamines. Understanding that DI may not represent a single, uniform disease or diagnostic entity and can have various underlying causes is essential for accurate diagnosis and effective treatment.

**Objectives:**

This study aims to examine the clinical presentation, etiology and therapeutic approach for a patient presenting with DI while comparing approaches for different DI etiologies.

**Methods:**

A case report was conducted on a 61-year-old woman presenting with delusion of infestation, skin lesions, depressive symptoms and a toxicology screening positive for cocaine. Addicionally, a literature review using the PubMed database was performed to identify relevant clinical articles on DI.

**Results:**

In evaluating the patient’s condition, the initial differential diagnosis included formication, a condition where patients experience sensations such as stinging or crawling on the skin without developing fixed delusions. Formication can arise from multiple neurological or systemic causes, including exposure to substances. A unique subtype of formication, known as “cocaine bugs,” is seen in cocaine users and produces similar tactile sensations. Cocaine-induced formication is often part of a broader cocaine-related delirium that involves hallucinations and ideas of contamination. For these patients, detoxification is a priority in treatment, addressing the substance use disorder directly. In cases where delusions develop alongside these sensations, particularly in individuals with pre-existing psychiatric conditions such as schizophrenia or depressive disorder, the diagnostic focus shifts.

**Conclusions:**

Delirium of infestation can occur in individuals with cocaine use and those with psychotic depression. More than half of the patients with DI have a history of depression and both conditions often coexist. There are individuals for whom DI appears to cause depression and patients for whom depression appears to precipitate DI. This case highlights the complexity in diagnosing and treating DI, particularly when cocaine use and psychotic depression co-occur. The patient’s symptoms reflect overlapping etiologies, with cocaine use likely exacerbating formication, while psychotic depression contributed to fixed beliefs of infestation. Given the frequent co-occurrence of DI and depressive symptoms, further research is needed to refine neurobiological insights and treatment strategies for DI across these patient groups.

**Disclosure of Interest:**

None Declared

